# Research on the Short-Circuit Characteristics of Trench-Type SiC Power MOSFETs Under Single and Repetitive Pulse Strikes

**DOI:** 10.3390/mi16070768

**Published:** 2025-06-29

**Authors:** Li Liu, Bo Pang, Siqiao Li, Yulu Zhen, Gangpeng Li

**Affiliations:** 1 State Key Laboratory of Wide-Bandgap Semiconductor Devices and Integrated Technology, School of Microelectronics, Xidian University, Xi’an 710071, China; ligp@stu.xidian.edu.cn; 2 Guangzhou Institute of Technology, Xidian University, Guangzhou 510555, China; pb2147705204@163.com (B.P.); leesq123@163.com (S.L.); zhenyulu9245@163.com (Y.Z.)

**Keywords:** SiC MOSFETs, trench gate, short circuit, degradation

## Abstract

This paper investigates the short-circuit characteristics of 1.2 kV symmetrical and asymmetrical trench-gate SiC MOSFETs. Based on the self-designed short-circuit test platform, single and repetitive short-circuit tests were carried out to characterize the short-circuit capability of the devices under different electrical stresses through the short-circuit withstanding time (SCWT). Notably, the asymmetric trench structure exhibited a superior short-circuit capability under identical test conditions, achieving a longer SCWT compared to its symmetrical counterpart. Moreover, TCAD was used to model the two devices and fit the short-circuit current waveforms to study the difference in short-circuit characteristics under different conditions. For the degradation of the devices after repetitive short-circuit stresses, repetitive short-circuit pulse experiments were conducted for the two groove structures separately. The asymmetric trench devices show a positive Vth drift, increasing on-resistance, increasing C_gs_ and C_ds_, and decreasing C_gd_, while the symmetric trench devices show a negative Vth drift, decreasing on-resistance, and inverse variation in capacitance parameters. Both blocking voltages are degraded, but the gate-source leakage current remains low, indicating that the gate oxide has not yet been damaged.

## 1. Introduction

Silicon carbide (SiC) has a higher breakdown electric field, thermal conductivity, and electron mobility than silicon (Si) material [1]. Its wide bandgap provides a higher operating temperature and higher breakdown electric field, which facilitates the reduction in chip size, capacitance, and on-resistance, its higher thermal conductivity contributes to better heat dissipation and lower thermal resistance, and its higher electron mobility contributes to a faster switching capability and higher current density. As a result, power electronic systems using SiC devices are more compact, efficient, and lightweight compared to the Si insulated gate bipolar transistor (IGBT) [2,3,4]. Meanwhile, SiC is the optimal choice for power devices, with its forbidden bandwidth, high breakdown electric field, thermal conductivity, and high frequency. This is especially the case in the field of electric vehicles, rail transportation, photovoltaic power generation systems, and wind power generation equipment [5]. Although SiC metal-oxide-semiconductor field-effect transistors (MOSFETs) are superior to Si MOSFETs in some cases, the reliability of SiC MOSFET devices is not ideal, especially under extreme conditions such as short-circuits, when the device is in a short-circuit state, and high drain-source voltages and saturation currents are applied to the device at the same time; these conditions result in SiC power MOSFETs with a very high transient power dissipation [6], which can lead to device degradation or even failure. It has been found that the typical short-circuit withstanding time (SCWT) of commercial SiC MOSFET devices is within 2–7 µs, and even shorter at high temperatures and high voltages [7].

Studying the effect of experimental conditions on the short-circuit reliability of devices can provide a reference for the application and protection of devices. Several teams at home and abroad have experimentally verified that device burnout caused by thermal runaway is the main reason for the failure of planar-gate SiC MOSFETs, and charge injection into the gate oxide layer is the mechanism that leads to the threshold voltage (Vth) drop. At present, the short-circuit characteristics of SiC MOSFETs are mainly studied in two directions: single short-circuit and repetitive short-circuit. For example, the short-circuit capability of three commercial 1200 V SiC MOSFETs at different temperatures and DC bus voltages were tested in [8]. It was found that the high temperature inside the device accelerates the generation of intrinsic carriers, which in turn further aggravates the junction temperature increase, forming a positive feedback loop that may eventually trigger thermal runaway burnout or gate oxide damage failure. In [9], the failure mechanism of the device was studied by high-speed optical images, which revealed the occurrence of thermal runaway failure. Jiaxing Wei’s team has comprehensively studied and analyzed the failure mechanism of double-trench (DT) SiC MOSFETs [10]. In addition, the effect of bias voltage on the short-circuit tolerance of the device has also been investigated, showing that the higher the gate and drain voltages, the shorter the short-circuit tolerance time. Xiaochuan Deng et al. proposed the prediction of the short-circuit capability and failure modes for double-trench and asymmetric (AT) structured SiC MOSFETs under the single pulse short-circuit stress [11] and analyzed the failure of the AT-MOSFET mode as the gate damage at a low drain voltage and thermal runaway at a high drain voltage, while DT-MOSFETs exhibited thermal runaway failure at all DC bus voltages. In [12], Jiaxing Wei et al. carried out a detailed study on the degradation of electrical parameters of planar-gate SiC MOSFETs under repetitive SC stress, and found that the injection of negative charges at the interface of the gate oxide layer in the trench region is the main degradation cause of the device’s Vth and on-resistance elevation at low gate voltages. Due to the positive shift in Vth and Miller’s plateau, the gate-source charge increases, the on-time and switching energy increases, the off-time is shortened, and the off-energy decreases. Xiaoqing Jia et al. investigated the degradation of different gate oxide regions of a planar-gate device under different repetitive DC bus voltages of a SiC MOSFET, and verified the above conclusions by using technology computer-aided design (TCAD) simulations, showing that under high DC bus voltages, the junction temperature and electric current of the SiC MOSFETs, with a significantly higher junction temperature and electric field, lead to the degradation of the oxide layer in the JFET region [13]. Dr. Renze Yu’s team investigated the short-circuit reliability of planar, double-trench, and asymmetric trench SiC MOSFETs under repetitive short-circuit pulses of 300 K and 450 K. The TCAD modeling of the three structures revealed that the degradation of the gate dielectric medium leads to the degradation of the structural parameters of the trench [14].

The rest of this paper is organized as follows. Section 2 describes the device structure and experimental setup. The structure and electrical characteristics of double-trench and asymmetric trench-type power SiC MOSFET devices are presented. The short-circuit schematic and typical short-circuit test waveforms are given. Section 3 investigates the single short-circuit characteristics of trench-type SiC MOSFET devices. Different gate-source voltages, drain-source voltages, and temperatures are chosen to experimentally test the short-circuit characteristics of the two commercial devices, and the effects of different conditions on the short-circuit withstand time of the devices are analyzed. The TCAD tool is utilized to further reveal the influence of the distribution of electro-thermal parameters inside the device on the short-circuit characteristics under different test conditions. In Section 4, repetitive short-circuit tests are performed on the devices by choosing different short-circuit times to record the degradation of static and dynamic parameters of the devices and analyze the causes of the degradation in combination with the TCAD tool. Finally, Section 5 summarizes the paper.

## 2. Device Structures and Experimental Setup

**Figure 1:**
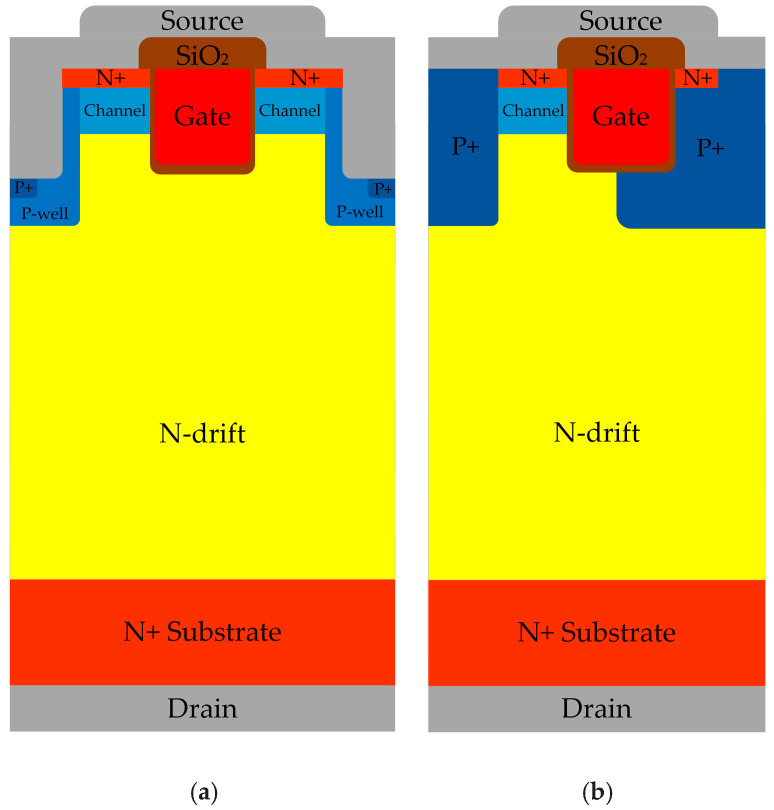
Cross-section of 4H-SiC trench MOSFET: (**a**) DT-MOSFET; (**b**) AT-MOSFET.

The devices under test (DUT) are from Rohm and Infineon. Figure 1a shows the schematic of the Rohm SCT3080KR dual-trench structure with a rated voltage of 1200 V, rated current of 31 A, on-resistance of 80 mΩ, and Vth of 4.1 V [15] manufactured in Kyoto, Japan. Figure 1b shows the schematic of the Infineon IMW120R090M1H asymmetric trench-gate structure, with a rated voltage of 1200 V, rated current of 26 A, on-resistance of 90 mΩ, and Vth of 4.5 V [16] manufactured in Neubiberg, Germany. All packages are TO-247-4L.

Both devices have similar current ratings and on-resistance, which can be used as a comparative study. Some of the electrical characteristics were tested before the short-circuit test experiment, as shown in Table 1, and the process consistency is good.

**Figure 2:**
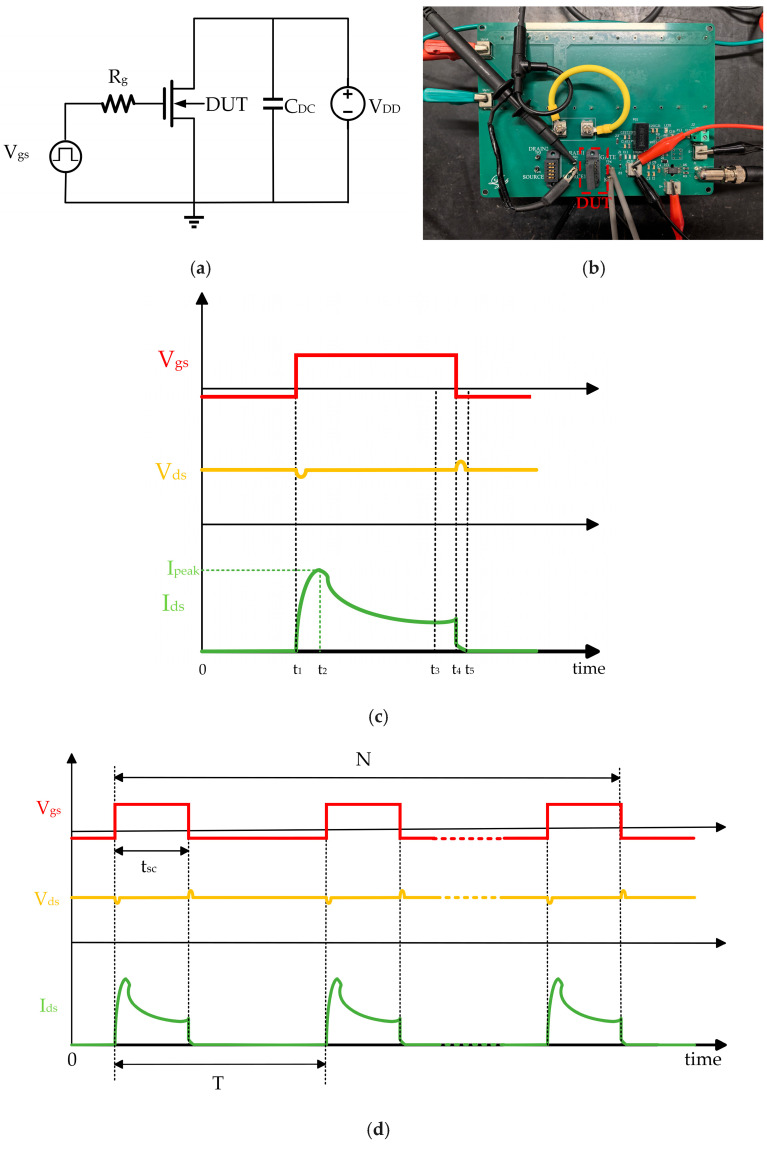
Short-circuit test of 4H-SiC power MOSFET: (**a**) schematic circuit of SC test; (**b**) SC test setup; (**c**) wave form diagram of single short-circuit test; and (**d**) wave form diagram of repetitive short-circuit test.

A common short-circuit test schematic is shown in Figure 2a. This test circuit diagram consists of the gate voltage (V_gs_), gate resistance (R_g_), and DUT to form the driving circuit. The voltage source (V_DD_), DC-stabilizing capacitor (C_DC_), and the short-circuit current are much larger than those of IGBT devices or IGBT modules. An IGBT can be connected in series to form a power supply circuit, and act as a circuit breaker in the test circuit; by controlling the DUT and the gate signal of the IGBT, a short-circuit fault can be deliberately triggered. This allows the DUTs and the main circuit to be disconnected after the fault occurs to enable a post-fault analysis. Figure 2c shows the hard-switched short-circuit waveforms for this circuit. This includes the gate drive voltage V_ge_ of the IGBT, drain-source voltage V_ds_, drain-source current I_ds_, and short-circuit peak current I_peak_. During the time period t_0_-t_1_, the IGBT is turned on, the drain-source pole of the device to be tested is subjected to the supply voltage V_DD_, and at the moment of t_1_, the DUTs conduct a short circuit. In order to ensure that most of the supply voltage is applied to the DUTs without affecting the results of the short-circuit test, the devices to be tested in the short circuit are set in the saturation region. Therefore, the IGBT used in the actual test should have a current level that is much higher than that of the devices to be tested. During the test, the IGBT is delayed for a period of time after the devices to be tested begin shutting down at moment t4, and it is only turned off at moment t6 to disconnect the power loop. This delay prevents the devices from being burned.

The repetitive short-circuit test has the same circuit topology as the single short-circuit test; the difference is that in the repetitive short-circuit test, the gate drive of the DUT is a repetitive pulse signal, and the test waveform is shown in Figure 2d. In order to test the degradation of device parameters without the failure of the device, the repeat short-circuit pulse width t_sc_ is usually chosen to be less than the short-circuit withstanding time of the device, and at the same time, the appropriate period T is chosen to ensure that the device is sufficiently dissipated after each short circuit to reduce the accumulation in the junction temperature on the parameter degradation of the impact of the junction temperature. Notably, as the spikes in V_ds_ are related to the L_loop_dI_ds_/dt, L_loop_ is the stray inductance in the power loop, and I_ds_ is the saturation current of the DUT; when I_ds_ starts to increase, a downward spike voltage occur between source and drain, while when I_ds_ is shut off, a overshoot spike V_ds_ occurs. During our experimental setup, to avoid noise disturbance and meet the large volume energy storage, three types of capacitors are adopted in parallel with the DC link to maintain the spikes in a controllable way. Figure 2b shows the short-circuit test setup, which can perform single short-circuit and repetitive short-circuit tests.

## 3. Single Short-Circuit Test Analysis of Trench Power SiC MOSFETs

### 3.1. Single Short-Circuit Test

#### 3.1.1. Effect of Gate-Source Voltage

First, the effects of different gate-source voltages on the short-circuit characteristics of the two trench devices were experimentally tested. The gate-source driving voltages were selected as 15 V, 16 V, and 18 V, respectively, and the effects of the three gate-source voltages on the short-circuit withstanding time under the short-circuit stress of the devices at a room temperature of 25 °C and voltage source of 400 V were tested. The pulse time of each gate-source voltage was gradually increased from 6 μs during the experiment, and in order to avoid the influence of the junction temperature increase during the device short circuit on the next short-circuit test, the next short-circuit test was carried out at an interval of 1 μs after the completion of each test. The test waveforms are recorded after each experiment until the devices fail or the three-terminal impedance is significantly smaller, and the test is stopped after the device fails.

**Figure 3:**
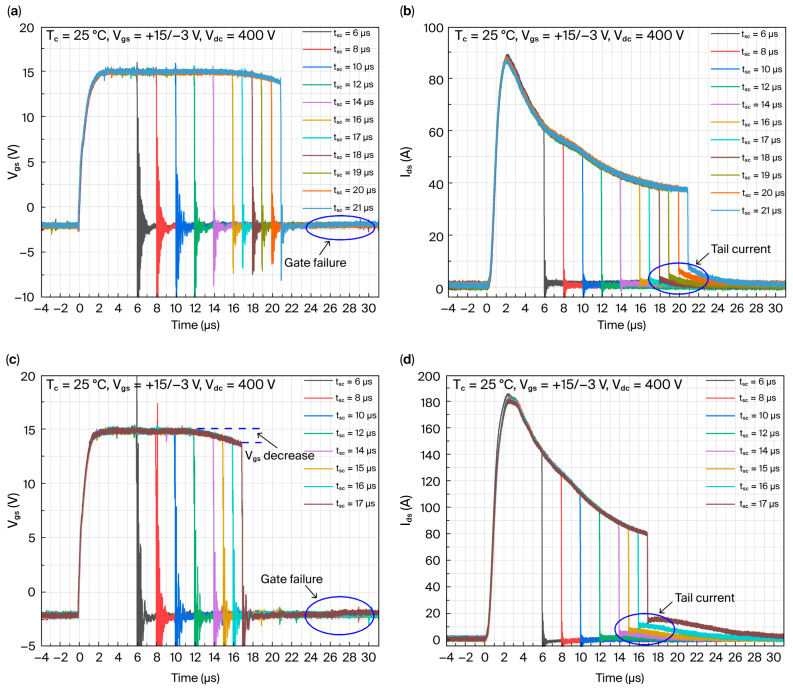
Short-circuit waveforms for AT-MOSFETs and DT-MOSFETs at gate voltage of 15 V: (**a**) Vgs of AT-MOSFETs; (**b**) Ids of AT-MOSFETs; (**c**) Vgs of DT-MOSFETs; and (**d**) Ids of DT-MOSFETs.

The short-circuit test plots of the AT-MOSFETs and DT-MOSFETs at the room temperature of 25 °C, voltage source of 400 V, and gate-source voltage of 15 V/−3 V are shown in Figure 3. From Figure 3a,c, it can be seen that the gate voltage of both trench devices decrease with the gradual increase in the short-circuit pulse duration. The AT-MOSFETs under a short-circuit stress with a gate pulse of 21 μs shows a gate voltage drop of 1.29 V at the 21st μs, whereas the DT-MOSFETs under short-circuit stress with a gate pulse of 17 μs shows a gate voltage drop of 1.4 V at the 17th μs. The peak short-circuit current of the AT-MOSFETs device in Figure 3b is 88.9 A, and the trail current occurs with the increase in short-circuit pulse time and gradually increases with the increase in short-circuit time. With a short-circuit time of 21 μs, the tail current after device shutdown is 7.9 A. After the experimental measurement, the impedance between the gate source of the device is 115.2 Ω, which indicates that the device has lost its blocking capability between the gate source and is judged to have failed. Under this short-circuit stress, the maximum short-circuit withstand time of the AT-MOSFETs is 21 μs, and the short-circuit critical energy is calculated to be 0.4306 J. Meanwhile, the peak short-circuit current of the DT-MOSFETs in Figure 3d is 178 A, and the trend of the change is the same as that of the AT-MOSFETs with the increase in the short-circuit pulse time. When the short-circuit time is 17 μs, the tail current after device shutdown is 14 A, and the tail current disappears 13 μs after device shutdown. At the end of the experiment, the impedance between the gate and the source of the device is measured to be 1592 Ω. The device also loses the blocking capability between the gate and the source and is judged to have failed. Under this short-circuit stress, the maximum short-circuit withstand time of the DT-MOSFETs is 17 μs, and the calculated short-circuit critical energy is 0.7984 J. The maximum short-circuit withstand time of the DT-MOSFETs is 17 μs.

In this case, the peak short-circuit current of the devices at a gate-source voltage 16 V/−3 V is 94.5 A, which is slightly increased from the gate-source voltage of 15 V/−3 V. For the gate-source voltage of 18 V/−3 V, under the short-circuit stress with a gate pulse of 18 μs, the device decreases the gate voltage by 1.39 V at the 18th μs, and the gate voltage starts to rise at the 3rd μs after the device is turned off, and the device fails with a final rise in the gate voltage of 0.64 V.

The short-circuit waveforms of the AT-MOSFETs and DT-MOSFETs at gate-source voltages of 16 V/−3 V and 18 V/−3 V are similar to those at gate-source voltages of 15 V/−3 V at room temperature of 25 °C and a voltage source of 400 V. Therefore, Table 2 is used to represent their various parameters and failures. For the DT-MOSFETs, at a gate-source voltage of 16 V/−3 V, the tail current gradually appears and increases. At a short-circuit time of 16 μs, the tail current is 16 A. After the device is turned off, the tail current increases slightly, and disappears after the device is turned off for 14 μs. When the gate-source voltage is 18 V/−3 V, the peak short-circuit current is 250 A, which is the largest among the three gate voltage tests; the tail current gradually increases, the tail current is 14.97 A after the device is turned off, the short-circuit time is 15 μs, and the tail current disappears 16 μs after the device is turned off.

**Figure 4:**
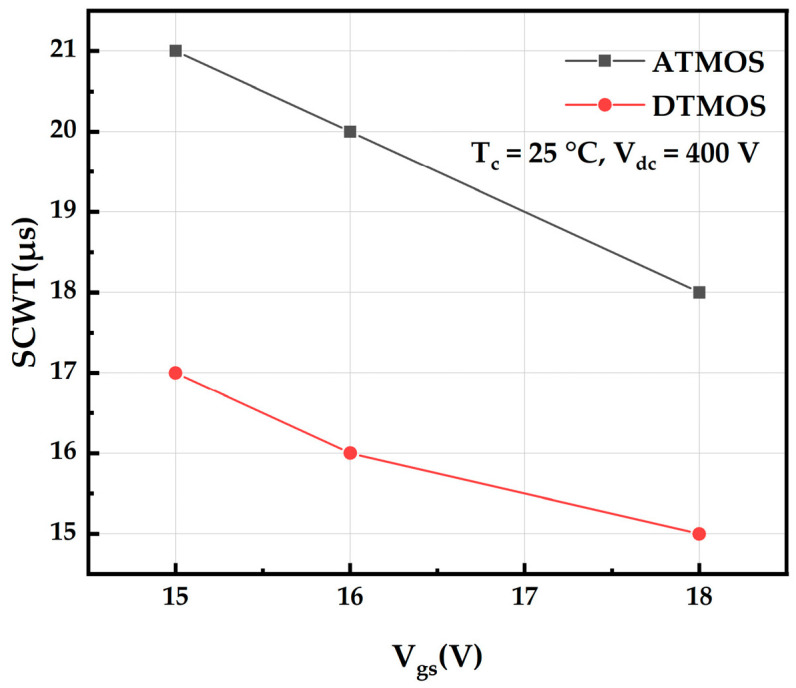
Short-circuit withstand time of trench-type SiC MOSFETs at different gate pressures.

Figure 4 shows the maximum short-circuit withstand time of AT-MOSFETs and DT-MOSFETs at the room temperature of 25 °C and the drain-source voltage of 400 V under different gate drive voltages. As the gate voltage increases, the peak short-circuit current of the device increases, leading to an increase in the energy dissipated in the device; further, the junction temperature rises faster, and failure is more likely to occur, thus having a shorter ultimate short-circuit withstand time. It has also been found that the short-circuit reliability of DT-MOSFETs is weaker than that of AT-MOSFETs [17].

#### 3.1.2. Effect of Temperature

Experiments were conducted to test the effect of different temperatures on the short-circuit characteristics of two trench devices. The temperatures were chosen to be 25 °C, 75 °C, and 125 °C, respectively, and the effects of the three temperatures on the short-circuit withstand time of the devices under the short-circuit stress of 400 V voltage source and 16 V/−3 V gate-source voltage were tested. During the experiment, the pulse duration of each gate-source voltage was gradually increased, starting from 6 μs. To avoid the influence of the increasing junction temperature caused by each short circuit, subsequent short-circuit tests were carried out with 1 μs intervals between them. The test waveforms are recorded after each test until the device fails or the three-terminal impedance becomes small, and the test is stopped at the point at which the device fails.

**Figure 5:**
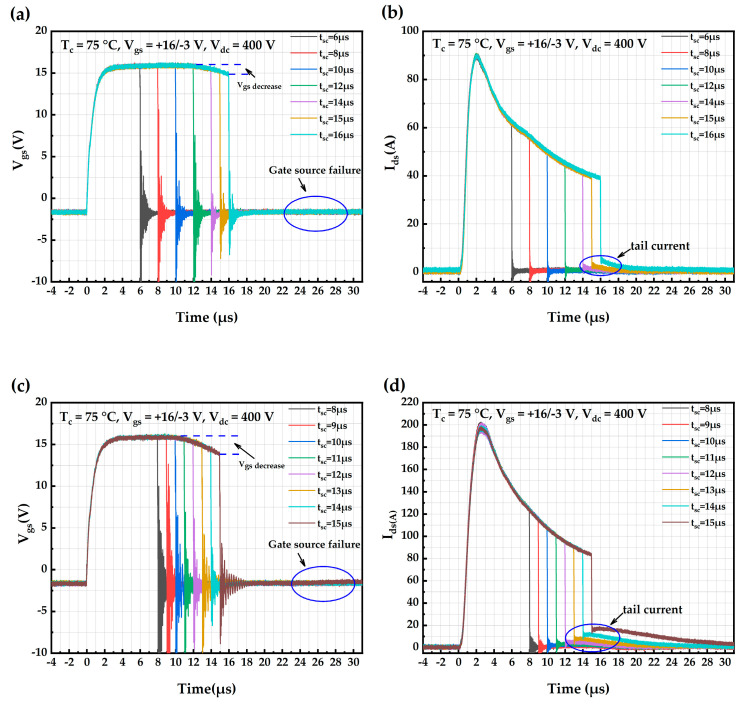
Short-circuit waveforms for AT-MOSFETs and DT-MOSFETs at 75 °C: (**a**) V_gs_ of AT-MOSFETs; (**b**) I_ds_ of AT-MOSFETs; (**c**) V_gs_ of DT-MOSFETs; and (**d**) I_ds_ of DT-MOSFETs.

Short-circuit test plots of AT-MOSFETs and DT-MOSFETs at 75 °C with a 400 V voltage source and 16 V/−3 V gate-source voltage are shown in Figure 5. In Figure 5a,c, the gate voltage decreases with the increasing short-circuit pulse duration. For AT-MOSFETs with a short-circuit stress of 16 μs for the gate pulse, the device’s gate voltage decreases by 1.19 V at the 16th μs. For DT-MOSFETs with a short-circuit stress of 15 μs for the gate pulse, the device’s gate voltage decreases by 2.1 V at the 15th μs. The peak short-circuit current of the AT-MOSFETs in Figure 5b is 89.3 A, which is lower than the short-circuit saturation current at 25 °C. As the short-circuit pulse time increases, a tail current occurs and becomes larger as the short-circuit time increases. With a short-circuit time of 16 μs, the tail current of the device after shutdown is 5.2 A. At the end of the experiment, the impedance between the gate and the source of the device is measured to be 668 Ω, which indicates that the device has lost the blocking ability between the gate and the source, and it is judged that the device has failed. Under this short-circuit stress, the maximum short-circuit withstand time of the AT-MOSFETs is 16 μs, and the short-circuit critical energy is calculated to be 0.3548 J. The short-circuit peak current of the DT-MOSFETs in Figure 5d is 197.6 A, which is lower than the short-circuit saturation current at 25 °C. As with the AT-MOSFETs, the trail current appears gradually and becomes larger. With a short-circuit time of 15 μs, the trail current after device shutdown is 15.9 A. At the end of the experiment, the impedance between the gate and the source of the device is measured to be 21.4 Ω, and the device has lost its blocking capability between the gate and the source; therefore, the device is judged to have failed. Under this short-circuit stress, the maximum short-circuit withstand time of the DT-MOSFETs is 15 μs, and the calculated short-circuit critical energy is 0.7454 J.

The three short-circuit waveforms of the AT-MOSFETs and DT-MOSFETs are similar for 25 °C, 75 °C, and 125 °C at a 400 V source and 16 V/−3 V gate source. Therefore, Table 3 represents their various parameters and failures. The peak short-circuit current of the AT-MOSFETs at 125 °C is 85.5 A, which is a further reduction compared to that at 75 °C.

**Figure 6:**
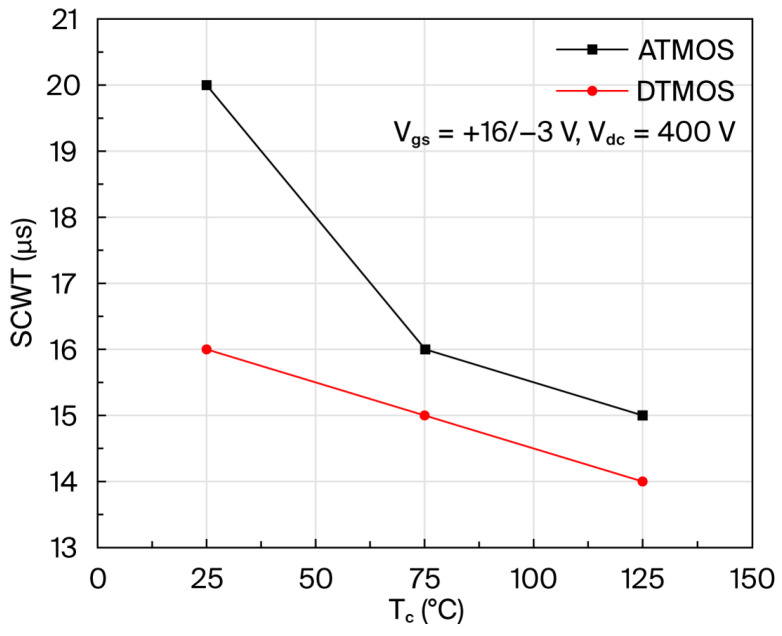
Short-circuit withstand time of trench-type SiC MOSFETs at different temperatures.

Figure 6 shows the maximum short-circuit withstand time for AT-MOSFETs and DT-MOSFETs at a drain-source voltage of 400 V and a gate-source voltage of 16 V/−3 V at case temperature. As the ambient temperature increases, the device junction temperature is higher, resulting in the device being more likely to reach the failure threshold junction temperature, resulting in device failure, and thus having a shorter ultimate short-circuit withstand time. The short-circuit reliability of AT-MOSFETs is also better than DT-MOSFETs, even at high temperatures [18].

#### 3.1.3. Effect of Drain-Source Voltage

The effect of short-circuit stress on the short-circuit withstand time and type of failure at 25 °C, voltage source of 600 V, and different gate-source voltages was tested. During the experiment, the pulse duration of each gate-source voltage was gradually increased from 4 μs, and in order to avoid the influence of the junction temperature increase on the next short-circuit test when the device is short-circuited, the next short-circuit test was carried out by increasing the pulse duration by 1 μs at an interval of time after the completion of each test. The test waveforms are recorded after the completion of each experiment, and the test is stopped when the device fails.

**Figure 7:**
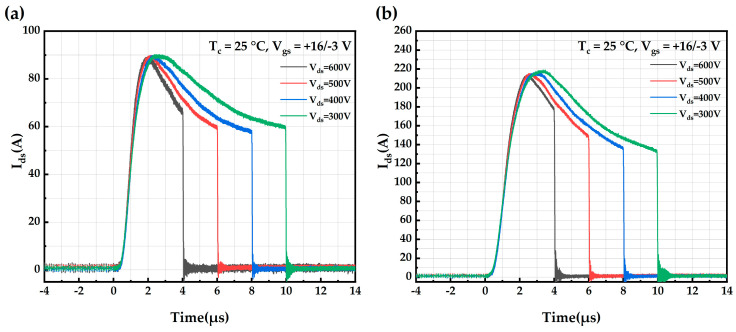
Short-circuit waveforms of trench-type SiC MOSFETs at different drain voltages: (**a**) AT-MOSFETs; (**b**) DT-MOSFETs.

Figure 7 shows the short-circuit current test waveforms of AT-MOSFETs and DT-MOSFETs at a room temperature of 25 °C and gate-source voltage of 16 V/−3 V at different drain voltages. As the drain voltage increases, the current increases and opens faster, but the short-circuit saturation current decreases.

**Figure 8:**
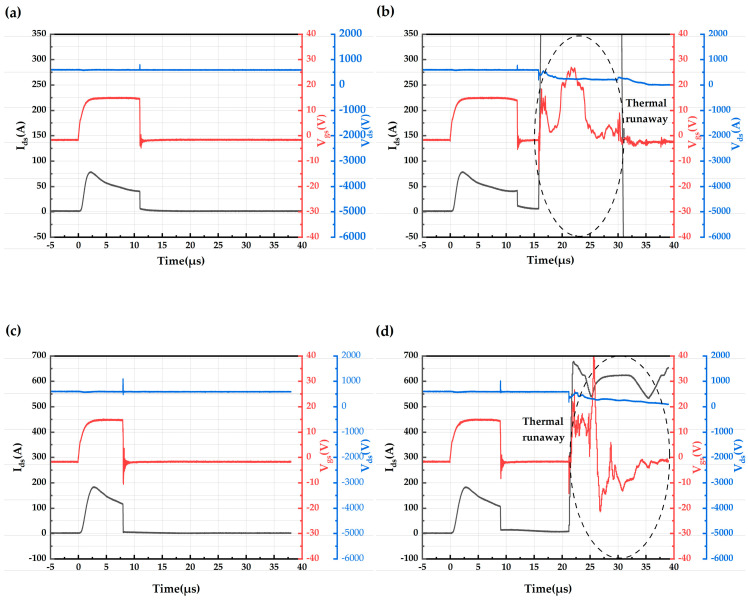
Short-circuit waveforms for AT-MOSFETs and DT-MOSFETs at drain voltage 600 V and gate voltage 15 V: (**a**) before failure waveform of AT-MOSFETs; (**b**) failure waveform of AT-MOSFETs; (**c**) before failure waveform of DT-MOSFETs; and (**d**) failure waveform of DT-MOSFETs.

The short-circuit test plot of the AT-MOSFETs and DT-MOSFETs at a room temperature of 25 °C, voltage source of 600 V, and gate-source voltage of 15 V/−3 V is shown in Figure 8. Figure 8a,c show the last test waveform graph before failure, and Figure 8b,d show the failure waveform. The black waveform in the figure is the short-circuit current, the red waveform is the gate-source voltage, and the blue waveform is the drain-source voltage. In Figure 8a, the drain-source current rises rapidly to 78.9 A after the AT-MOSFETs are turned on, and then the current begins to drop. As the short-circuit time increases, the gate voltage appears to drop. When the device is turned off at the 11th μs, the gate voltage drops by 0.6 V and the trail current is 6.59 A. The drain-source voltage spikes during device turn-on and turn-off due to the presence of stray inductance in the power loop. In Figure 8b, as the short-circuit time increases, the current first rises to the peak short-circuit current and then begins to decrease, with the increase in short-circuit current occurring after the 11th μs. When the device is turned off, the gate voltage drops by 1.2 V at the 12th μs, which is an increase over the pre-fault short-circuit stress. The trail current is 12.35 A, which is also an increase over the pre-failure short-circuit stress. After the device was turned off for 3.9 μs, the current increased dramatically, and at the same time, the device gate was out of control, and the drain-source voltage gradually dropped to 0 V, indicating that the device lost its blocking capability, which is a typical thermal runaway failure. The maximum short-circuit withstand time of the AT-MOSFETs under this short-circuit stress is thus obtained to be 12 μs.

In Figure 8c, the drain-source current rises rapidly to 184 amps after the DT-MOSFETs are turned on, after which the current begins to drop. As the short-circuit time increases, the gate voltage shows a drop. When the device is turned off at the 8th μs, the gate voltage drops by 0.3 V and the trail current is 7.29 A. Due to the presence of stray inductance in the power loop, the drain-source voltage spikes during the time the device is turned on and off. In Figure 8d, as the short-circuit time increases, the current first rises to the peak short-circuit current and then begins to decrease. At the 9th μs of device turn-off, the gate voltage drops by 0.7 V, which is an increase over the pre-fault short-circuit stress. The trail current is 14.1 A, which is also increased compared with the pre-failure short-circuit stress, and the tail current lasts until 21.2 μs. After the device is turned off for 12.2 μs, the current increases dramatically, and at the same time, the device gate goes out of control, which indicates that the device loses its blocking capability, and this is a typical thermal runaway failure. It can be seen that the maximum short-circuit withstand time of the DT-MOSFETs is 9 μs under this short-circuit stress.

The short-circuit test waveforms of AT-MOSFETs with gate-source voltages of 18 V/−3 V and 16 V/−3 V are similar to the short-circuit test plots of 15 V/−3 V at a room temperature of 25 °C and voltage source of 600 V. Therefore, Table 4 is used to represent their various parameters and failures. T_gate failure_ is the time after device shutdown when the current increases dramatically while the device gate is out of control. Both trench devices exhibit thermal runaway failures.

**Figure 9:**
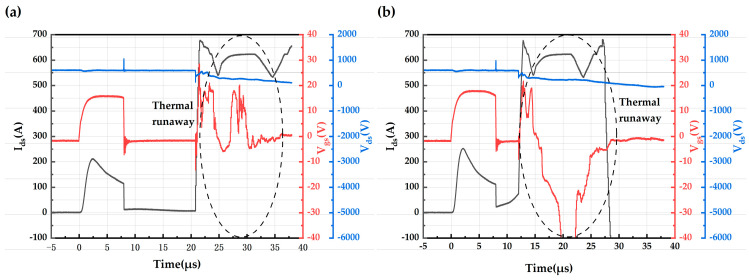
Short-circuit failure waveforms for DT-MOSFETs at drain voltage 600 V, V_gs_ 16 V and 18 V: (**a**) 16 V; (**b**) 18 V.

The device exhibits a thermal runaway failure under all three stresses. Figure 9 shows the short-circuit failure waveforms of the DT-MOSFETs at a drain voltage of 600 V and gate voltages of 16 V and 18 V. The gate voltage decreases by 0.8 V when the device is turned off at the 8th μs in Figure 9a, which is an increase from the pre-failure short-circuit stress. The tail current is 12.7 A, which is also increased from the pre-failure short-circuit stress, and the trail current lasts until 20.8 μs. In Figure 9b, the gate voltage decreases by 2.0 V when the device is turned off at 8 μs, which is increased from the pre-failure short-circuit stress. The tail current is 22.6 A, which is also increased from the pre-failure short-circuit stress, and the trail current continues to increase up to 12 μs, which is different from the gradual decrease in the trail current under low gate voltage stress. The current increases sharply after the device is turned off for 4 μs, while the device gate is out of control, and the drain-source voltage gradually decreases to 0 V [11].

**Figure 10:**
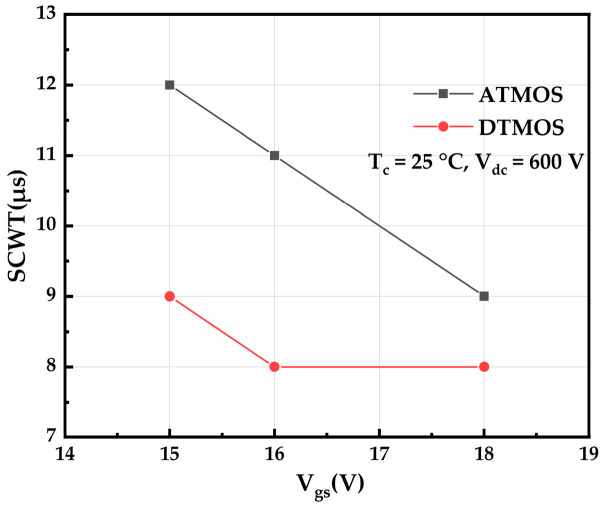
Short-circuit withstand time for trench-type SiC MOSFETs at a drain voltage of 600 V.

Figure 10 shows the maximum short-circuit withstand time of AT-MOSFETs and DT-MOSFETs at a room temperature of 25 °C and drain-source voltage of 600 V for different gate drive voltages. The ultimate short-circuit withstand time of the devices is shorter as the gate voltage increases, which is consistent with the trend at the voltage source of 400 V. It is also found that the short-circuit withstand time of the device is shorter at a high drain voltage and the failure mechanism is completely different from that at a low drain voltage. The short-circuit robustness of the DT-MOSFETs is still lower than that of the AT-MOSFETs at a high voltage source [19,20].

### 3.2. TCAD Simulation Under Single Short-Circuit Testing

The key parameters of typical DT-MOSFET and AT-MOSFET TCAD models are shown in Table 5. The thickness of the substrate is used to represent the full model of the device and does not represent the actual thickness. Physical models, including the avalanche generation model, “Okuto-Crowell Model”, Shockley–Read–Hall (SRH) recombination model, temperature and high electric field-dependent carrier mobility model, incomplete ionization thermodynamics model, and temperature-dependent intrinsic carrier density model are taken into consideration in the simulation. It is worth noting that the decreasing trend of the current after reaching I_peak_ is controlled by the lattice temperature. The temperature coefficients of thermal conductivity and thermal capacity are introduced to accurately describe the lattice temperature, and at the same time, in order to better characterize its electro-thermal phenomenon, its thermal conductivity and thermal capacity can be referred to [21].

Although there are differences between the model parameters and the actual device, the distribution results of the electrical and thermal parameters of the simulation model are still representative and can provide a reference for analyzing the short-circuit characteristics of the device.

**Figure 11:**
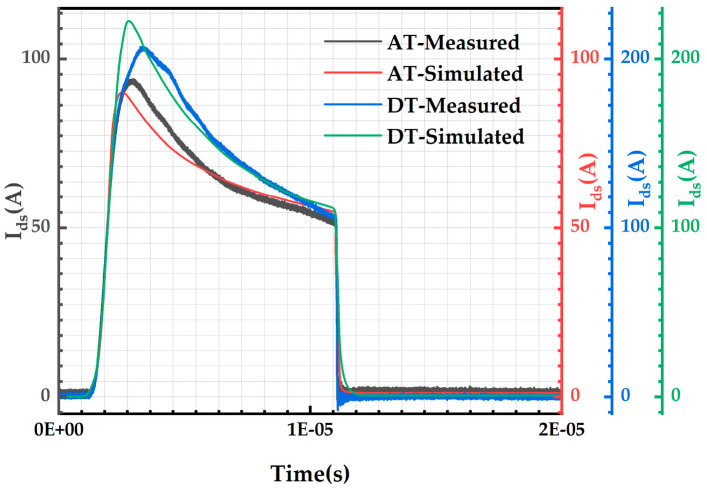
Comparison of experimental and simulated short-circuit currents for TCAD with AT-MOSFETs and DT-MOSFETs structure.

For the AT-MOSFETs, the gate-source voltage of 16 V, the drain-source voltage of 400 V, the temperature of 300 K, and the short-circuit time of 10 μs are selected, and the fitted single short-circuit experimental current waveforms are shown as the red and black waveforms in Figure 11. For DT-MOSFETs, the same simulation conditions as AT-MOSFETs and a short-circuit time of 10 μs are chosen, and the fitted single short-circuit experimental current waveforms are shown as the blue and green waveforms in Figure 11. From the comparison graph of experimental waveforms and simulation results, it can be seen that the two are in good agreement; although there are differences, the overall trend is similar, and this can be used as a general case study. Based on this model, we observe the effect of different test conditions on the distribution of the internal electrical and thermal parameters of the device and analyze the reasons for the failure of the device.

**Figure 12:**
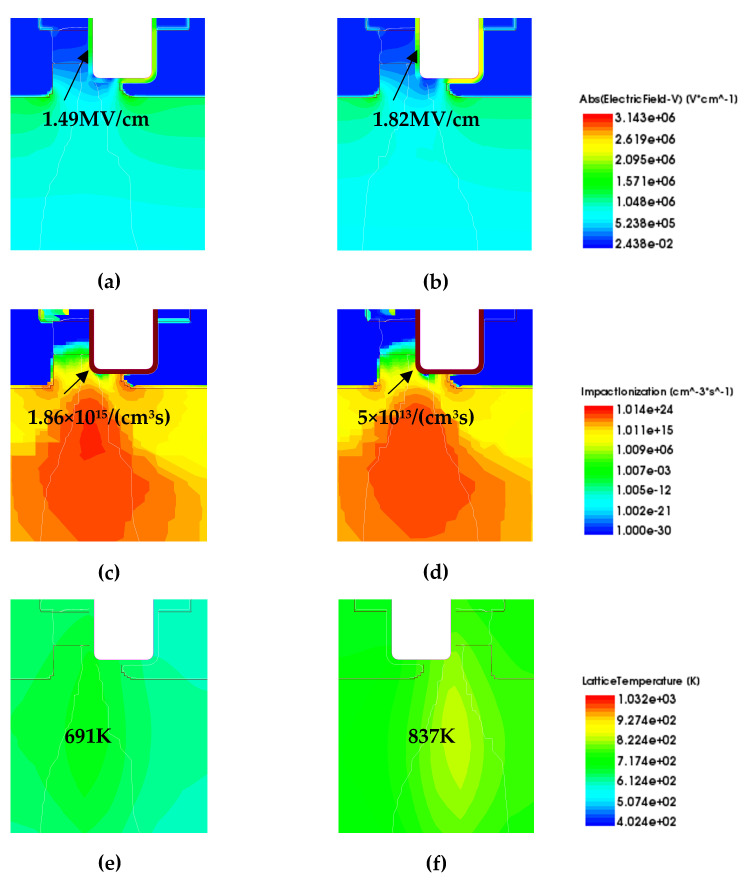
Electric field, impact ionization rate, and lattice temperature distributions of AT-MOSFETs at different gate pressures. Electric field distribution: (**a**) 15 V and (**b**) 18 V; impact ionization rate distribution: (**c**) 15 V and (**d**) 18 V; lattice temperature distribution: (**e**) 15 V and (**f**) 18 V.

Figure 12 shows the distribution of electric field intensity, impact ionization rate, and lattice temperature of the AT-MOSFETs after 2 μs of short-circuit turn-on, with different gate-source voltages at a drain-source voltage of 400 V and a temperature of 300 K. Basically, the peak point of the short-circuit current is shown in Figure 3b,d. It can be seen that the electric field at the trench-gate oxide (E_trench-gate oxide_) becomes larger with the increase in the gate voltage, and the impact ionization rate decreases at the corners of the trench (α_corners_). After the same short-circuit time, the lattice temperature of the device is also higher under high gate pressure conditions. The gate voltage affects the on-resistance, which in turn affects the peak short-circuit current, junction temperature, and short-circuit withstand time [22]. At a gate voltage of 18 V, the device has the highest junction temperature and the worst condition and therefore has the shortest short-circuit withstand time [22], a result that is consistent with the experiment. The short-circuit withstand time is shorter than that of the 16 V and 15 V cases, but the junction temperature of the 18 V case in Figure 12 is higher than that of the 15 V case.

**Figure 13:**
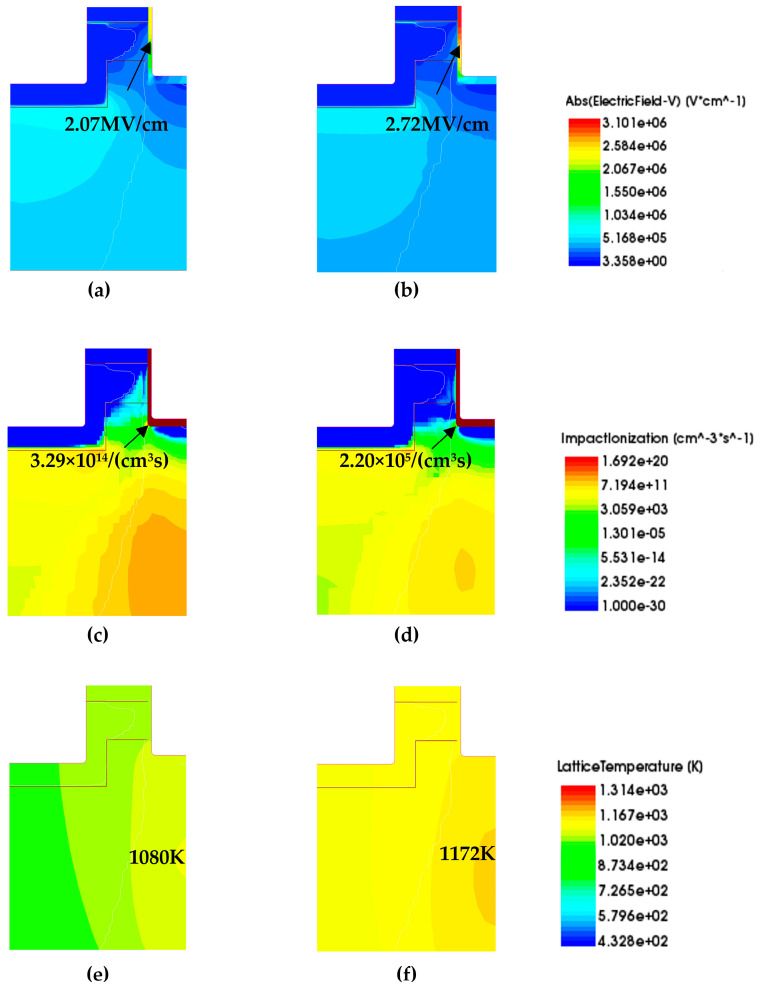
Electric field, impact ionization rate, and lattice temperature distributions of DT-MOSFETs at different gate pressures. Electric field distribution: (**a**) 15 V and (**b**) 18 V; impact ionization rate distribution: (**c**) 15 V and (**d**) 18 V; lattice temperature distribution: (**e**) 15 V and (**f**) 18 V.

Figure 13 shows the distributions of the electric field, impact ionization rate, and lattice temperature of the DT-MOSFETs after short-circuit conduction for 2 μs with different gate-source voltages at a drain-source voltage of 400 V and a temperature of 300 K. The temperature of the trench devices is also higher at the higher gate voltage for the same short-circuit time. The short-circuit withstand time of the device is the shortest at a gate voltage of 18 V, which is consistent with the experimental results.

Under the above simulation method, we extracted the maximum values of the electric field, maximum values of the impact ionization rate, and maximum values of the lattice temperature for the two trench devices under different operating conditions (different V_gs_, different temperature, and different V_ds_) as shown in Table 6, and the operating conditions are still extracted under the maximum value of current I_peak_ at 2 μs. The electric field at the gate oxide of the AT-MOSFETs is almost unaffected by temperature at different temperatures, while the impact ionization rate at the corners of the trench is slightly reduced. The short-circuit withstand time is shortest at higher ambient temperatures where the lattice temperature of the device is also higher, a finding that agrees with the experimental results. The variation in the gate oxide electric field with gate voltage at 300 K and drain voltage of 600 V for different gate voltages is the same as that at a drain voltage of 400 V. This is because the gate oxide electric field is mainly related to the gate voltage, which is slightly lower than that at the drain voltage of 400 V. The higher the gate voltage, the lower the impact ionization rate at the trench corners, which is the same as that at low drain-source voltages, but the value of the impact ionization rate is much higher than at low drain-source voltages. The junction temperature of the device rises faster at 600 V and the lattice temperature is higher. This harsher test condition also results in a shorter short-circuit withstand time, which is the same as the experimental results.

The gate-oxide electric field and temperature dependence of the DT-MOSFETs at 2 μs after short-circuit conduction are the same as those of the AT-MOSFETs under different temperature conditions with a gate voltage of 16 V and a drain-source voltage of 400 V. The DT-MOSFETs are also found to have a higher initial junction temperature than the AT-MOSFETs. The higher initial junction temperature makes the device easier to reach the failure condition, which is consistent with the experimental results. In addition, the gate oxide electric field of the DT-MOSFETs is slightly lower than that at a drain-source voltage of 400 V, and the impact ionization rate at the trench corners is higher at a temperature of 300 K and a drain-source voltage of 600 V after short-circuited conduction for 2 μs at different gate voltages. The junction temperature accumulates in a very short time and the device is prone to destructive failure, leading to a shortened short-circuit withstand time, which is the same as the experimental findings.

### 3.3. Failure Analysis Under Single Short-Circuit Testing

The single short-circuit experiments of the two trench-type devices revealed that the manifestation of device failure was different when the bus voltage was different: under the test condition of a 400 V bus voltage, with the increase in the short-circuit pulse time, the gate voltage was gradually reduced, the tail current appeared and gradually increased, and finally, the device was able to shut down safely. The decrease in gate voltage is caused by the gate-source leakage current (I_gss_), and the tail current contains components such as the thermal excitation current and avalanche excitation current [23]. From the simulation, it can be seen that the internal temperature of the device increases rapidly during the short-circuit process, and when the electrons obtain enough energy, they can cross the interface between SiC and SiO_2_ to form an I_gss_ under the action of the electric field to cause the gate voltage reduction during the short-circuit process. These phenomena are indicative of the eventual failure of the device. At the same time, whether at room temperature or higher temperatures, the device always shows a consistent failure phenomenon under the bus voltage of 400 V test conditions, and the phenomenon of the rising gate voltage after the device is turned off or the low resistance of the device gate source at the end of the test is also called the gate-source failure mode. Under test conditions with a bus voltage of 600 V, the device eventually fails to shut down and burns out when it reaches the limit short-circuit withstand time, i.e., thermal runaway failure mode.

**Figure 14:**
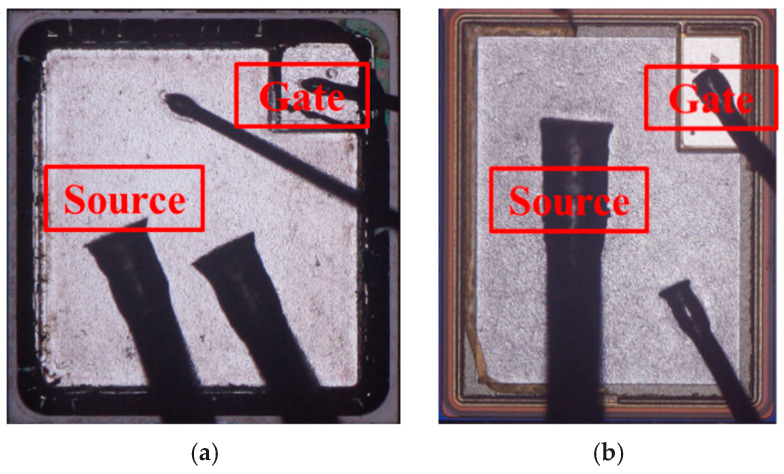
De-encapsulation results after short-circuit failure of trench-type SiC MOSFETs at 400 V drain voltage: (**a**) AT-MOSFET; (**b**) DT-MOSFET.

Figure 14 shows the depackaged plots of two trench-type devices after a short-circuit failure at a 400 V drain-source voltage, as evidenced by the gate failures labeled in Figure 3a,c. The trend is insignificant for V_gs_ of 15 V, but there is a clear trend in the gate voltage from 3 V to 0 V for the 18 V gate failure. There are almost no visible burnt spots on the chip surface, which is consistent with the impedance values tested after the experiment and also indicates that the gate damage occurs in the internal gate oxide layer of the device [24]. Upon the arrival of the short-circuit condition, the increased junction temperature of the device leads to an increase in the thermal stress between the aluminum metal and the gate oxide layer. When the electro-thermal stress reaches the tolerance limit of the gate oxide, it leads to SiO_2_ cracking. As the short circuit progresses, after the temperature continues to rise and is sufficient to melt the aluminum metal, the molten aluminum penetrates into the gate oxide cracks, causing the gate source to fail [20]. The device short-circuit tolerance time is longer, and the temperature rise is slower at bus voltages with less stress, and the final temperature is not sufficient to generate a large number of electron–hole pairs, causing thermal runaway and exhibiting a gate failure mode [25].

**Figure 15:**
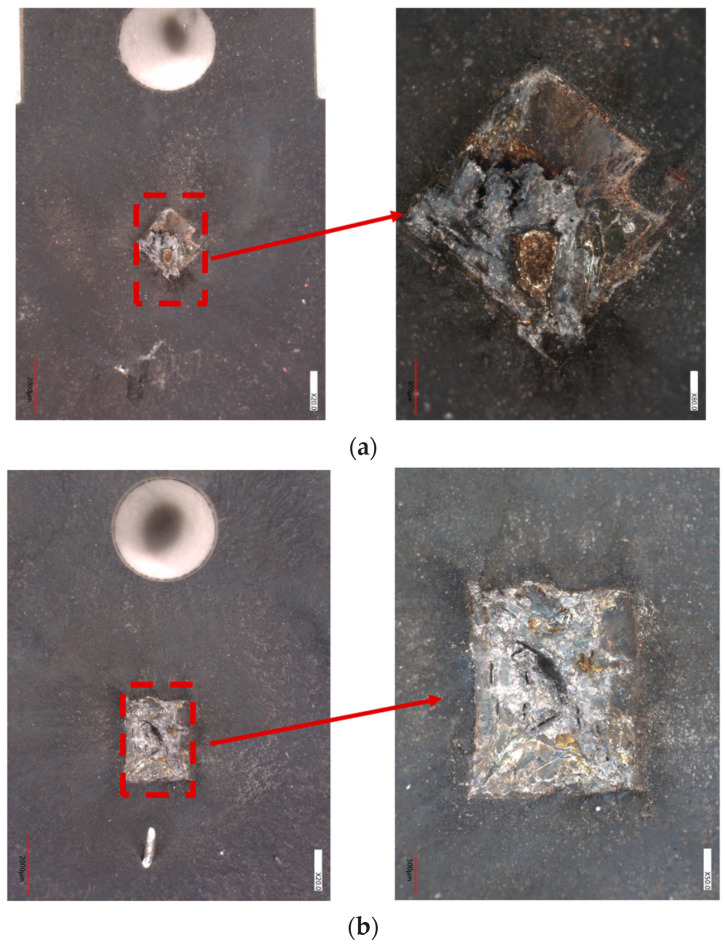
Surface view of the device after short-circuit failure of a trench-type SiC MOSFET at 600 V drain voltage: (**a**) AT-MOSFET (V_gs_ = 18 V, 9 μs); (**b**) DT-MOSFET (V_gs_ = 18 V, 7.5 μs).

A surface view of two trench-type devices after short-circuit failure at the 600 V drain-source voltage is shown in Figure 15. This failure corresponds to the thermal runaway labeled in Figure 8b,d and Figure 9, as well as the tail current observed in the curves. The entire active region is severely burned, and the bonding wires are completely melted. Under a short circuit with a high drain-source voltage, a sharp temperature rise occurs inside the chip within only a few μs, and the high temperature leads to the generation of carriers in the drift region to form a tail current [26]. The high voltage and high temperature generate a large number of electron–hole pairs, which in turn increase the current density, causing the temperature of the device to continue to rise, and ultimately leading to thermal runaway failure, which is manifested by the trench-type device exploding during the experiment, with all of the aluminum metal on the surface of the chip melting and burning.

**Figure 16:**
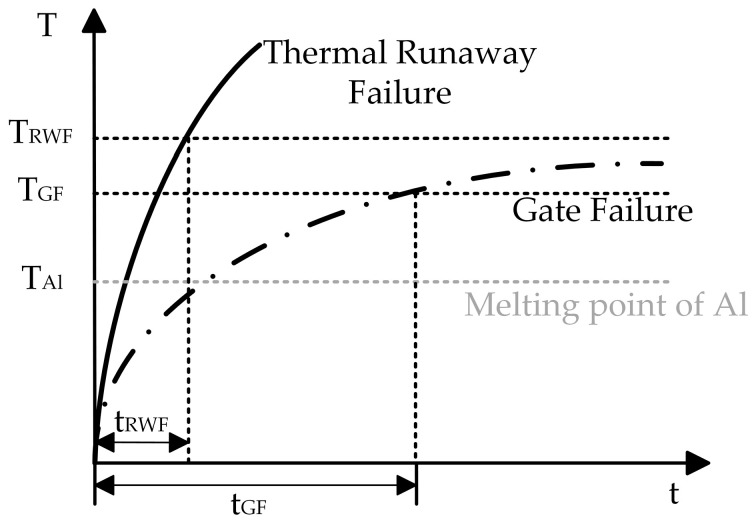
Short-circuit failure mechanism of trench-type devices [27].

The failure mechanism of trench-type devices under different short-circuit conditions is shown in Figure 16. When the junction temperature rises faster, generating a large number of carriers leads to an increase in the positive feedback of the current and temperature, which ultimately results in the thermal runaway failure of the device, and this critical temperature is referred to as T_RWF_. However, when the junction temperature rises slowly, there is enough time to build up electro-thermo-mechanical stresses, and once the temperature is high enough (T_GF_) to melt the Al, the melted Al gets into the broken oxide layer and causes the gate to short out. T_Al_ in the figure is the critical temperature for Al melting.

## 4. Repetitive Short-Circuit Testing of Trench Power SiC MOSFETs

### 4.1. Repetitive Short-Circuit Test

In order to study the degradation of static and dynamic parameters of trench-type SiC MOSFET devices after short-circuit stress, the short-circuit test experimental program is repeated to avoid the accumulation of heat on the next cycle of short-circuit, and the final choice of the bus voltage is 400 V, the gate-source voltage is 16 V/−3 V, the gate pulse frequency selection is 2 Hz, and the duty cycle is 0.001%. The degradation of the device can be observed after a certain period of time under this short-circuit stress and does not lead to rapid failure. By setting a pulse period of 500 ms and adding heat sinks and air cooling to the device under test, multiple methods are used to ensure the heat dissipation of the device. In addition, a pulse frequency of 2 Hz and a duty cycle of 0.002% were chosen to compare the degradation and even failure of the device parameters, because 10 μs is the typical short-circuit time of power devices in operation. The static and dynamic parameters of the device are tested after selecting the appropriate number of repetitive short circuits to study the degradation. Trench-type SiC MOSFET devices may fail after repetitive short-circuit stress. When the I_gss_ increases due to damage to the gate oxide layer to the extent that the device is unable to perform switching control, it is determined to be a failure, and the experiment is terminated.

**Figure 17:**
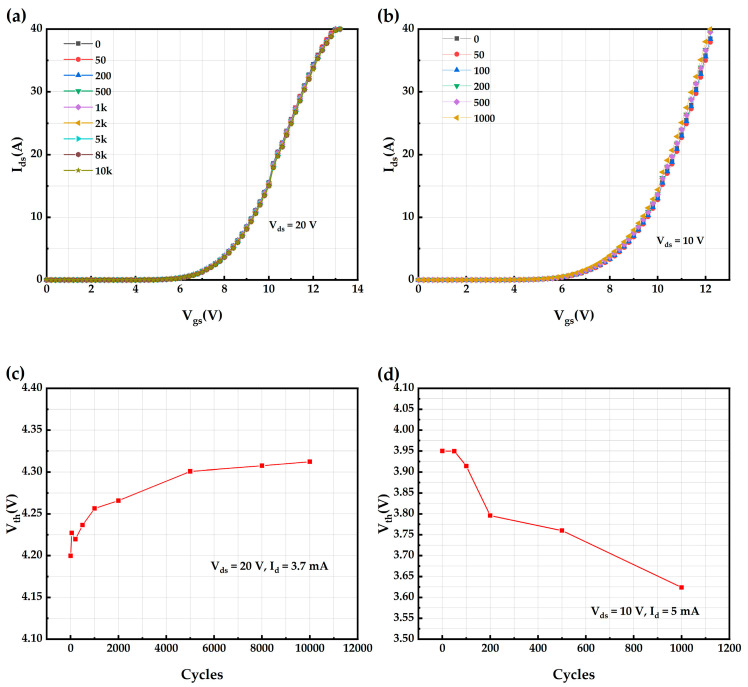
AT-MOSFETs and DT-MOSFETs under 5 μs short-circuit pulse: (**a**) transfer characteristics of AT-MOSFETs; (**b**) transfer characteristics of DT-MOSFETs; (**c**) Vth of AT-MOSFETs; and (**d**) Vth of DT-MOSFETs.

The degradation of the transfer characteristics of the AT-MOSFETs under repetitive short-circuit tests with 5 μs short-circuit pulses is shown in Figure 17a, from which it can be seen that the transfer characteristics of the device under this repetitive short-circuit stress shifts slightly to the right with the increase in the number of short-circuits. The degradation of the transfer characteristics and Vth of the DT-MOSFETs under repetitive short-circuit tests with 5 μs short-circuit pulses is shown in Figure 17b, and the device fails after about 1100 repetitive SC stresses before the device fails. It can be seen that the transfer characteristic of the device under this repetitive short-circuit stress shifts to the left with the increase in the number of short circuits, implying the degradation of the Vth. Figure 17c shows the variation of the Vth of the device (extracted at V_ds_ = 20 V, I_d_ = 3.7 mA, T_c_ = 25 °C) with the number of short circuits. Its value shows an increase from the initial 4.20 V to 4.31 V. Consistent with several previous reports, all Vth values of the SC-stressed device show a similar increasing degradation trend [28,29,30]. It is believed that both V_gs_on_ and massive electrons [29,30] trapped in the gate oxide generated by channel collisional ionization during the short circuit through the coupled electric field will result in an increase in electrons in the gate oxide. When a higher V_gs_on_ of 16 V is applied to the gate, electrons trapped at the corner of the trench will weaken the collection of electrons, thus affecting its current carrying capability, which can be seen in Figures 10 and 11 in ref. [29]. Consequently, the Vth will increase. Figure 17d shows the variation in the Vth (extracted at V_ds_ = 10 V, I_d_ = 5 mA, T_c_ = 25 °C) of the DT-MOSFETs with the number of short circuits. The value shows a decreasing trend from the initial 3.95 V to 3.62 V. In agreement with previous reports [14,31,32], all SC-stressed devices show a similar degradation decreasing trend in the Vth. This is in contrast to the degradation trend of the AT-MOSFETs under 5 μs repetitive short-circuit pulses. This is due to the fact that the residual high temperature after the previous short-circuit strike causes a large number of charged holes generated by channel collisional ionization to overcome the barrier and become injected into the gate oxide. When the negative V_gs_off_ lasts longer, more holes are injected into the gate oxide, ultimately counteracting and outweighing the effect of the injected electrons generated during previous on-state strike, which can be seen in Figures 20 and 21 in ref. [14]. After undergoing the same number of SC stresses, the degradation of the Vth of the DT-MOSFETs is higher than that of the AT-MOSFETs.

**Figure 18:**
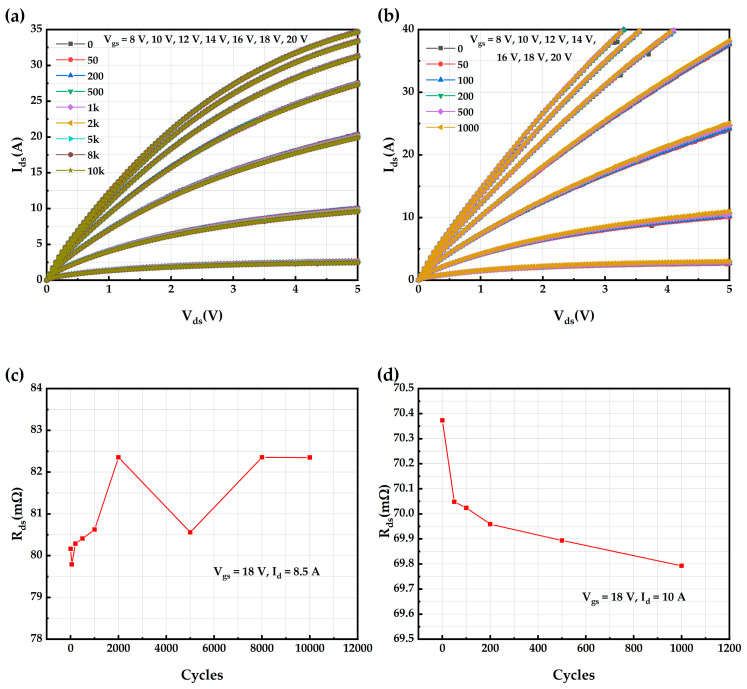
AT-MOSFETs and DT-MOSFETs under 5 μs short-circuit pulse: (**a**) output characteristic curve of AT-MOSFETs; (**b**) output characteristic curve of DT-MOSFETs; (**c**) R_ds_ of AT-MOSFETs; and (**d**) R_ds_ of DT-MOSFETs.

The variation in the output characteristic curve of the AT-MOSFET under a repetitive short-circuit test with 5 μs short-circuit pulses is shown in Figure 18a. From this, it can be observed that the output curve shifts slightly downward as the number of short-circuits increase. Figure 18c shows the variation in the on-resistance R_ds_ of the AT-MOSFETs (extracted at V_gs_ = 18 V, I_d_ = 8.5 A, T_c_ = 25 °C), with the number of short-circuits. The on-resistance also shows an overall increasing trend with the increase in short-circuit stress, from an initial 80.16 mΩ to 82.35 mΩ. This is due to an increase in Vth, a decrease in the effective repetitive voltage applied to the device channel, and a downward shift in the output characteristic curve [33]. Meanwhile, the output characteristic curve of the DT-MOSFETs under short-circuit tests with 5 μs short-circuit pulses changes, as shown in Figure 18b. It can be seen that the output curve variation is the same as that of the AT-MOSFETs, and the on-resistance decreases gradually. Figure 18d shows the variation in the on-resistance R_ds_ of the DT-MOSFETs (extracted at V_gs_ = 18 V, I_d_ = 10 A, and T_c_ = 25 °C) with the number of short circuits. The R_ds_ gradually decreases with the increase in short-circuit stress, from the initial 70.37 mΩ to 69.79 mΩ. This is due to the decrease in the Vth, which results in an increase in the effective voltage applied to the device channel, and is the result of the upward shift of the output characteristic curve [33,34].

**Figure 19:**
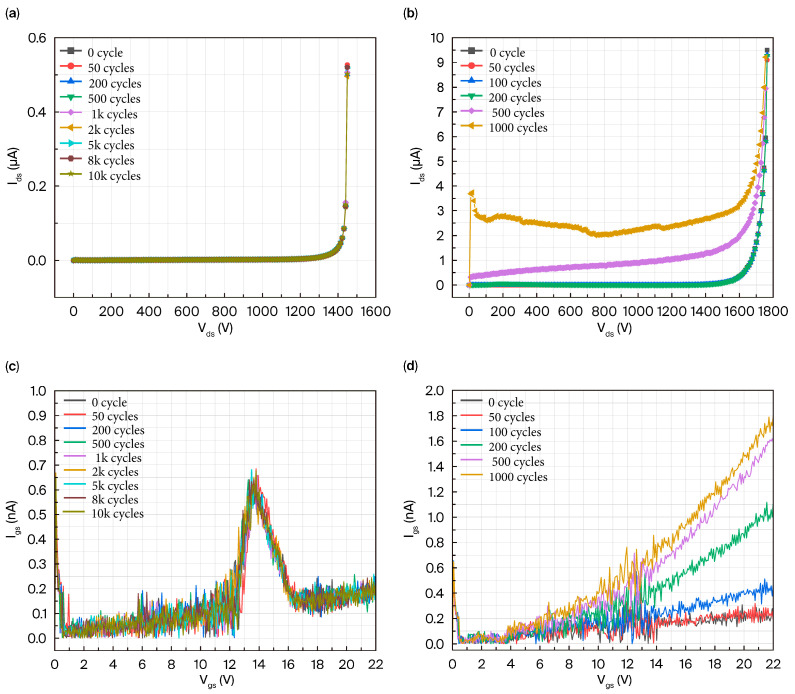
AT-MOSFETs and DT-MOSFETs under 5 μs short-circuit pulse: (**a**) blocking characteristics of AT-MOSFETs; (**b**) blocking characteristics of DT-MOSFETs; (**c**) I_gss_ of AT-MOSFETs; and (**d**) I_gss_ of DT-MOSFETs.

The blocking characteristics of the AT-MOSFETs under repetitive short-circuit testing with 5 μs short-circuit pulses are shown in Figure 19a. The blocking characteristics remain almost unchanged, even at the 10 k short-circuit stress. The trench-type SiC MOSFET relies heavily on the body diode reverse blocking withstand voltage, so it is shown that the body diode blocking of the device does not degrade significantly under this stress. Figure 19c shows the variation in the I_gss_ of the AT-MOSFETs with the number of short circuits. In order to be consistent with the datasheet, the I_gss_ is extracted at V_ds_ = 0 V and V_gs_ = 22 V. It is found that the I_gss_ of the AT-MOSFETs is always maintained below 0.3 nA after repetitive short-circuit tests, which indicates that the gate oxide of the device is not damaged, and the degradation is not obvious. Figure 19b shows the variation in the blocking characteristics of the DT-MOSFETs under repetitive short-circuit tests with 5 μs short-circuit pulses. Under 200 repetitions of short-circuit stress, there is no significant change in the blocking characteristics, while under 500 repetitions of the short-circuit stress, the blocking voltage decreases until the device almost loses its reverse blocking capability under 1000 repetitions of short-circuit stress and finally fails. Under this stress, the blocking capability of the device body diode is severely degraded. The I_gss_ is extracted at V_ds_ = 0 V and V_gs_ = 22 V, and it is found that the I_gss_ of the DT-MOSFETs becomes larger with the number of short-circuits after repetitive short-circuit tests, increasing from 0.22 nA to 1.75 nA. The gate-source leakage current remains at a low level, indicating that the gate oxygen of the device is not damaged, but the degradation is more evident than in the AT-MOSFETs. Their trends are similar to those of [14,28,35,36].

**Figure 20:**
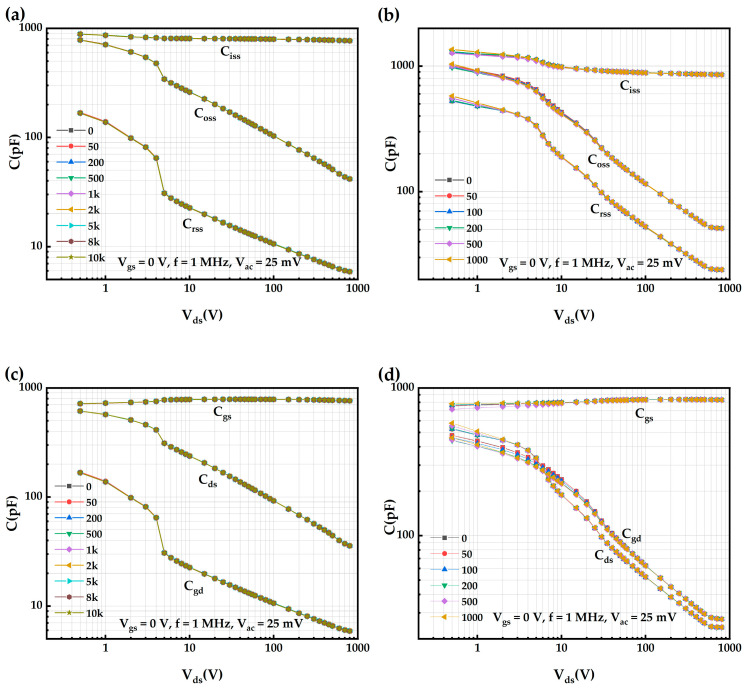
AT-MOSFETs and DT-MOSFETs under 5 μs short-circuit pulse: (**a**) C_iss_, C_oss_, C_rss_ of AT-MOSFETs; (**b**) C_iss_, C_oss_, C_rss_ of DT-MOSFETs; (**c**) C_gs_, C_ds_, C_gd_ of AT-MOSFETs; and (**d**) C_gs_, C_ds_, C_gd_ of DT-MOSFETs.

The degradation of the input capacitance (C_iss_), output capacitance (C_oss_), and reverse transfer capacitance (C_rss_) of AT-MOSFETs obtained from testing at V_gs_ = 0 V, f = 1 MHz, and V_ac_ = 25 mV after a repetitive short-circuit testing with 5 μs short-circuit pulses is shown in Figure 20a. The capacitance did not degrade significantly, and the capacitance was extracted at V_ds_ = 800 V, where the C_iss_ increased from the initial 769.143 pF to 769.853 pF after 10k SC stresses. Moreover, the C_oss_ changed from 41.593 pF to 41.569 pF, and the C_rss_ changed from 5.907 pF to 5.881 pF. Therefore, none of them showed obvious signs of degradation. Meanwhile, the C_gs_, C_ds_, and C_gd_ of the device can be calculated from C_iss_, C_oss_, and C_rss_, and their degradation is shown in Figure 20c. It can be seen that the C_gs_ increases from the initial 763.236 pF to 763.972 pF after 10k SC stresses, the C_ds_ changes from 35.686 pF to 35.688 pF, and the C_gd_ changes from 5.907 pF to 5.881 pF, indicating no significant degradation was observed. However, for DT-MOSFETs, under repetitive short-circuit tests with 5 μs short-circuit pulses and the same test conditions as those for AT-MOSFETs, the capacitance curves show a gradual increase in input capacitance, output capacitance, and reverse transfer capacitance as the number of short-circuits is increased at low leakage voltages. The variation in capacitance decreases with the increase in leakage voltage. Extracting the capacitance values at V_ds_ = 800 V, the C_iss_ decreases from the initial 855.478 pF to 852.481 pF after 1k short-circuit stresses, the output capacitance C_oss_ decreases from 51.160 pF to 51.121 pF, and the reverse transfer capacitance C_rss_ increases from 23.897 pF to 23.976 pF, which has a clear degradation trend. Moreover, C_gs_ decreases from the initial 831.581 pF to 828.505 pF after 1k SC stresses, a decrease of 3.076 pF, C_ds_ decreases from 27.263 pF to 27.144 pF, a decrease of 0.119 pF, and C_gd_ increases from 23.897 pF to 23.976 pF, a 0.079 pF increase, with a significant degradation.

The transfer characteristics of AT-MOSFETs under repetitive short-circuit test conditions with 10 μs short-circuit pulses and the Vth extracted from the devices under the same conditions are consistent with the degradation trend at the 5 μs short-circuit pulse stress. Meanwhile, the output characteristic curve is shifted downward under the repetitive short-circuit test condition of the 10 μs short-circuit pulse, which is consistent with the trend at the 5 μs short-circuit pulse stress. The on-resistance R_ds_ also shows an increasing trend with the increase in short-circuit stress, from the initial 82.08 mΩ to 84.34 mΩ, and the degradation trend is the same as that at the 5 μs short-circuit pulse stress. The blocking characteristics of the AT-MOSFETs are almost unchanged under the repetitive short-circuit test with a 10 μs short-circuit pulse, even after 1000 times of short-circuit stress, with good reverse blocking characteristics. However, the blocking voltage decreases, and degradation occurs under 2000 times of short-circuit stress. Under 3000 times of short-circuit stress, the device still has a reverse blocking capability after shutdown, but the blocking voltage is obviously reduced and the device blocking characteristics are seriously degraded. The device finally failed after about 3410 repetitive short-circuit tests with 10 μs short-circuit pulses. Extracting the gate leakage current of V_gs_ at 22V gate-source voltage, the gate leakage current was found to be consistently below 0.3nA under 10μs repetitive short-circuit stress, indicating that the gate oxygen of the device was also not significantly damaged. Under the repetitive short-circuit test with a 10 μs short-circuit pulse, the degradation of the capacitance is greater than that of the 5 μs repetitive short-circuit stress, the C_iss_ becomes larger with the increase in the number of short-circuits, the C_rss_ decreases with the increase in the number of short-circuits, and the degradation of the C_oss_ is not obvious. Moreover, C_gs_ and C_ds_ increase and C_gd_ decreases, respectively, and the degradation trend is consistent with the results of the repetitive short-circuit test with a 5 μs short-circuit pulse.

The transfer characteristics of the DT-MOSFETs under repetitive short-circuit testing with 10 μs short-circuit pulses show little change with the increasing number of short-circuits, which is due to the fact that the device withstands a smaller number of short-circuit conditions, and the device fails after 310 times. The Vth of the device was extracted and found to decrease with the number of short circuits, from the initial 3.97 V to 3.93 V, which is consistent with the degradation trend at the 5 μs short-circuit pulse stress. With the increase in the number of short circuits, R_ds_ gradually decreases from the initial 70.95 mΩ to 70.29 mΩ, and the degradation trend is the same as that at the 5μs repetitive short-circuit pulse stress. The device almost loses its blocking capability after 300 repetitive SC stresses, after which the device fails. As the number of short-circuits increase, the gate-source leakage current I_gss_ of the DT-MOSFETs gradually increases under this repetitive short-circuit stress, but remains well below 100 nA, indicating that the gate oxygen of the device is also not significantly damaged. Under the repetitive short-circuit test with a 10 μs short-circuit pulse, the C_iss_ and C_oss_ decrease with the increase in the number of short-circuits, the C_rss_ increases with the increase in the number of short-circuits, C_gs_ and C_ds_ increase, and C_gd_ decreases, respectively. The degradation trend is consistent with the results of the repetitive short-circuit test with the 5 μs short-circuit pulse.

### 4.2. Failure Analysis Under Repetitive Short-Circuit Stresses

The degradation of some of the parameters of the two devices at the drain-source voltage of 400 V, gate voltage of 16 V, and repetitive short-circuit testing at 25 °C for 5 μs and 10 μs is shown in Table 7.

After repetitive short-circuit experiments, the Vth and on-resistance of the devices show a certain degree of degradation, and the I_gss_ of the DUTs all increase slightly during repetitive short-circuits but still remain in the nA order of magnitude, indicating that there is no damage to the oxides during repetitive short circuits. It is worth noting that the degradation of I_gss_ is more obvious for DT-MOSFETs than AT-MOSFETs under the same test conditions, which indicates that the gate oxides of DT-MOSFETs are more fragile [35]. For AT-MOSFETs under the 5 μs repetitive short-circuit test, the blocking voltage of the devices did not degrade significantly even after 10,000 short-circuit cycles, while under the more severe 10 μs short-circuit condition, the blocking voltage degraded after 3000 repetitions of short-circuit stress, and finally, the devices failed after about 3100 repetitions of the short-circuit test. For DT-MOSFETs, the blocking voltage degradation is even faster, with the blocking voltage decreasing significantly after 500 repetitions of the experiment under the 5 μs repetitive short-circuit test, and the blocking effect almost disappearing after 1000 repetitions of the experiment. Under the 10 μs test, serious degradation occurs after 300 short-circuit cycles. It can be seen that the blocking characteristics of the AT-MOSFETs are better than those of the DT-MOSFETs under short-circuit conditions.

Both trench-type devices show consistent degradation directions under 5 μs and 10 μs test conditions, respectively. For the AT-MOSFETs, the Vth becomes larger, the on-resistance increases, and both C_gs_ and C_ds_ increase while C_gd_ decreases. For DT-MOSFETs, the Vth becomes smaller, the on-resistance decreases, and both C_gs_ and C_ds_ decrease while C_gd_ increases. This is considered to be a reflection of Vth degradation.

The Vth of both trench-type devices exhibit divergent degradation trends. As for the irrational “−0.04 V”, which is obtained under a 10 μs pulse width in Table 7, as we know, 10 μs is close to the critical short-circuit withstanding time. If the pulse width is set to 10 μs, the DT-MOS fails after 310 cycles, while under a 5 μs pulse width, the device fails after 1100 cycles. Therefore, the absolute value of “−0.04 V” cannot be trusted, but the minus sign of “−0.04 V” can be conceivable, which is illustrated in Figure 17 and ref. [14]. During a short-circuit event, the positive gate bias drives electrons into the channel and sidewalls, where electron injection into the oxide layer and subsequent accumulation may induce a positive Vth shift. Upon the termination of the short-circuit process and device turn-off, while the device remains at elevated temperatures, holes generated through intense impact ionization at the gate oxide bottom under a negative gate bias acquire sufficient energy to penetrate the oxide layer. The trapped holes at the oxide bottom migrate toward the sidewalls, while stored electrons in the sidewalls drift downward. A prolonged negative gate bias, which in our test is a 99.99% duty ratio, facilitates the additional hole injection into the gate oxide, with accumulates holes, ultimately causing a negative Vth shift. Owing to their inherently higher interface defect density, trench-type devices demonstrate more pronounced Vth drift phenomena. Furthermore, the magnitude of the positive gate bias significantly influences the drift characteristics. At lower positive bias levels, the coupled electric field at the oxide bottom—resulting from the joint action of gate and drain potentials—directs toward the gate oxide, promoting hole injection and a consequent negative Vth drift. Consequently, in repetitive short-circuit tests conducted in this study, AT-MOSFETs exhibited enhanced electron injection into the gate oxide, leading to Vth enlargement, whereas DT-MOSFETs experienced a predominant hole injection, causing Vth reduction [31].

The degradation of on-resistance has a relationship with the change in Vth. From the degradation status of AT-MOSFET on-resistance, the on-resistance increases consistently with the increase in Vth, indicating that the channel resistance does not undergo significant degradation, and the increase in on-resistance is a reflection of the increase in Vth. As for the DT-MOSFETs, under the 10 μs repetitive short-circuit experiment, the degradation is not obvious, although the threshold value decreases gradually, but the degradation of the on-resistance with the Vth is more obvious relative to the 5 μs repetitive short-circuit experiment, and one explanation is that under the 10 μs short-circuit condition, the degradation of the gate oxide layer of the DT-MOSFETs is more serious, which leads to an increase in I_gss_, and the I_gss_ applied to the gate resistor R_g_ results in a lower effective voltage applied to the gate and a larger on-resistance.

Since the repetitive short-circuit experiments use a 400 V bus voltage, similar to the single short-circuit experiments, as the number of pulses increases, the accumulated electro-thermal and mechanical stresses will ultimately lead to the cracking of the gate oxide layer and formation of conductive channels between the gate sources, and ultimately, all of this will occur as a result of the gate-source failure.

## 5. Conclusions

In this paper, an in-depth study on the short-circuit reliability of 1.2 kV commercial AT-MOSFETs and DT-MOSFETs was carried out, and the single short-circuit characteristics and repetitive short-circuit characteristics of the devices were systematically investigated by means of theoretical analysis, experimental testing, and numerical simulation. The short-circuit test platform was utilized to carry out single short-circuit experimental tests on two types of trench-type SiC MOSFETs. Different gate pressures, ambient temperatures, and different gate voltages at the 600 V drain-source voltage were selected, and the short-circuit withstand times of the devices under different SC-stressed were finally obtained. The experimental results show that the higher the gate voltage, the higher the short-circuit saturation current of the device and the shorter the short-circuit withstand time. The higher the temperature, the shorter the short-circuit withstand time, although the short-circuit saturation current of the device decreases due to the temperature characteristics of the channel resistor, but the high temperature environment makes the device more likely to reach the failure temperature; thus, a shorter short-circuit withstand time is observed. At a drain-source voltage of 600 V, the device finally explodes, exhibiting a thermal runaway failure mode.

According to the limiting short-circuit withstand time, different short-circuit pulse times were selected for repetitive short-circuit experimental tests on both devices, and the degradation of device parameters was investigated by extracting parameters after each set of experiments. It was found that the Vth of the asymmetric trench shows a positive drift, on-resistance increases, C_gs_ and C_ds_ increases, and C_gd_ decreases. The symmetric trench devices, on the other hand, show the opposite degradation trend of a negative Vth drift, decreasing on-resistance, decreasing C_gs_ and C_ds_, and increasing C_gd_. The simulation analysis revealed that the devices have the same distribution pattern of electrical and thermal parameters for 5 μs and 10 μs short circuits. 

## Figures and Tables

**Table 1:** Static and capacitive characteristics of trench SiC MOSFET devices.

	IMW120R090M1H	SCT3080KR
Vth (V)	4.20~4.35	3.85~4.05
R_ds,on_ (mΩ)	79~83	70~72
V_sd_ (V)	3.89~3.93	3.12~3.19
I_gss_ (nA)	0.20~0.40	0.15~0.60
C_iss_ (nF)	0.7691~0.7823	0.8260~0.8425
C_oss_ (nF)	0.0415~0.0417	0.0511~0.0512
C_rss_ (nF)	0.0059~0.0061	0.0237~0.0240

**Table 2:** Summary of the effects of 15 V, 16 V, and 18 V gate-source voltages on AT-MOSFETs and DT-MOSFETs in a single short-circuit test.

Component	AT-MOSFET			DT-MOSFET		
V_gs_ (V)	15	16	18	15	16	18
Short-circuit time (μs)	21	20	18	17	16	15
I_g decrease_ (A)	1.29	0.92	1.39	1.4	1.8	2.8
I_peak_ (A)	88.9	94.5	102.7	174	204	250
I_trail_ (A)	7.9	7.9	9.3	14	16	14.97
R_gs_ (Ω)	1.1	1.8	2.8	1.5	1.9	3.2
Short-circuit critical energy (J)	0.4306	0.4327	0.3974	0.7984	0.7884	0.7972

**Table 3:** Summary of the effects of 25 °C, 75 °C, and 125 °C on AT-MOSFETs and DT-MOSFETs in a single short-circuit test.

Component	AT-MOSFET			DT-MOSFET		
Temperature (°C)	25	75	125	25	75	125
Short-circuit time (μs)	20	16	15	16	15	14
I_g decrease_ (A)	0.92	1.19	1	1.8	2.1	1.9
I_peak_ (A)	94.5	89.3	85.5	204	197.6	190.8
I_trail_ (A)	7.9	5.2	4.2	16	15.9	13.3
R_gs_ (Ω)	1.8	0.7	0.4	1.9	2.1	1.0
Short-circuit critical energy (J)	0.4327	0.3548	0.3253	0.7884	0.7454	0.6765

**Table 4:** Summary of the effect of different gate-source voltages on AT-MOSFETs and DT-MOSFETs at a voltage source of 600 V in a single short-circuit test.

		Before Failure Waveform				Failure Waveform			
Component	V_gs_ (V)	Time (μs)	V_g decrease_ (V)	I_ds increases_ (A)	I_trail_ (A)	V_g decrease_ (V)	I_trail_ (A)	SCWT_max_ (μs)	T_gate failure_ (μs)
AT-MOSFET	15	11	0.6	78.9	6.59	1.2	12.35	12	3.9
	16	10	1	87.9	9.18	2	17.8	11	9.85
	18	8	0.8	105.1	7.18	2.7	15.4	9	2.1
DT-MOSFET	15	8	0.3	184	7.29	0.7	14.1	9	12.2
	16	7	0.4	213	7.06	0.8	12.7	8	12.8
	18	7	0.7	252	7.76	2	22.6	8	4

**Table 5:** The key parameters of typical DT-MOSFETs and AT-MOSFETs models.

Component	AT-MOSFET	DT-MOSFET
Cell width (μm)	3	3.6
Gate oxide thickness (nm)	75	50 (sidewall), 100 (bottom)
Channel length (nm)	500	500
N-drift thicknesss (μm)	11	10
N+ Substrate thicknesss (μm)	1	1
Doping of N+ (cm^−3^)	1 × 10^20^	1 × 10^20^
Doping of Channel (cm^−3^)	5 × 10^17^	8 × 10^17^
Doping of P-well (cm^−3^)	5 × 10^18^	-
Doping of N-drift (cm^−3^)	1 × 10^16^	7.5 × 10^15^
Doping of N+ Substrate (cm^−3^)	1 × 10^20^	1 × 10^20^

**Table 6:** TCAD simulation parameters for AT-MOSFETs and DT-MOSFETs.

Component	Factor	-	E_trench-gate oxide_ (MV/cm)	α_corners_ (cm^−3^s^−1^)	T_lattice_ (K)
AT-MOSFET	V_gs(Vds 400 V)_	15 V	1.49	1.86 × 10^15^	691
		16 V	1.58	5.61 × 10^14^	747
		18 V	1.82	5.00 × 10^13^	837
	Temperature _(Vds 400 V, Vgs 16 V)_	300 K	1.584	5.61 × 10^14^	747
		350 K	1.588	2.00 × 10^14^	813
		400 K	1.604	1.02 × 10^14^	862
	V_gs(Vds 600 V)_	15 V	1.46	1.12 × 10^17^	912
		16 V	1.56	3.26 × 10^16^	958
		18 V	1.80	2.26 × 10^15^	1029
DT-MOSFET	V_gs(Vds 400 V)_	15 V	2.07	3.29 × 10^14^	1080
		16 V	2.27	3.29 × 10^11^	1127
		18 V	2.72	2.20 × 10^5^	1172
	Temperature _(Vds 400 V, Vgs 16 V)_	300 K	2.27	3.29 × 10^11^	1127
		350 K	2.30	2.22 × 10^11^	1162
		400 K	2.32	8.76 × 10^10^	1182
	V_gs(Vds 600 V)_	15 V	2.04	6.45 × 10^18^	1261
		16 V	2.23	7.09 × 10^16^	1289
		18 V	2.65	4.14 × 10^15^	1309

**Table 7:** Summary of degradation of repetitive short-circuit test parameters.

Component	AT-MOSFET				DT-MOSFET			
Parameters	ΔV_th_	ΔR_ds_	C_gs,_C_ds_	C_gd_	ΔV_th_	ΔR_ds_	C_gs,_C_ds_	C_gd_
5 μs	+0.11 V	+2.19 mΩ	↑ *	↓ *	−0.33 V	−0.58 mΩ	↓	↑
10 μs	+0.12 V	+2.26 mΩ	↑	↓	−0.04 V	−0.66 mΩ	↓	↑

*: ↑ denotes increasing between 0 cycle and the final test before failure, ↓ denotes decreasing between 0 cycle and the final test before failure.

## Data Availability

The original contributions presented in this study are included in the article. Further inquiries can be directed to the corresponding author.
